# Supplementary material for: Screening and biodiversity analysis of cultivable inorganic phosphate–solubilizing bacteria in the rhizosphere of Hydrilla verticillata
Source: PLoS One. 2024 Jan 19;19(1):e0297047. doi: 10.1371/journal.pone.0297047 (PMC10798520; doi:10.1371/journal.pone.0297047)
Supplement: Attachment — Submitted filename: Response to Reviewers.docx [file pone.0297047.s001.docx]

Reviewer 1

1. The introduction is a little long, and the article's goal is poorly written.

Reply: Accepted and they have been corrected.

2. In the section on material and methods, why did the authors ignore measuring the pH during the quantification test?

Reply: We measured the pH of the culture solution during the experiment, which has been added to the three chapters of Materials and methods, Results and discussion and Conclusions.

3. In the Section 3.2, (In the future, IPB with better phosphate-solubilizing………. the experiments were set up as untreated ) Did you carry out this experiment, or did you suggest perspectives?

Reply: These are our perspectives. They have been corrected in the manuscript.

4. There are various grammatical errors in the paper that should be fixed.

Reply: Accepted and they have been corrected.

Reviewer 2

1. There are some grammatical errors throughout the paper; they need to be corrected.

Reply: Accepted and they have been corrected.

2. In section 2.4, authors should add a part in which they explain how they are calculating Phosphate solubilizing Efficiency.

Reply: Accepted and it has been added.

3. In section 2.4, the quantitative test of phosphate solubilization is always accompanied by a drop in pH, therefore why the authors ignored measuring it during the test?

Reply: We measured the pH of the culture solution during the experiment, which has been added to the three chapters of Materials and methods, Results and discussion and Conclusions.

4. In the introduction, Lines 78 to 83 must be moved to the discussion; the introduction should provide a clear rationale for the research and create a solid foundation for the following sections.

Reply: Accepted and they have been corrected.

5. In section 3.2, figure 2 is a repetition of column 3 of Table 2, the authors can remove it since it does not give new results.

Reply: Accepted and it has been deleted.

6. In section 3.2, from line 295 to line 304, the author gives perspectives but the content of the text is the past “In the future, IPB with better phosphate-solubilizing……. The experiments were set up as untreated and treated H……”

Reply: Accepted and they have been corrected.

7. In general, the section results and discussion must be reorganized to eliminate some repetitions.

Reply: Accepted and they have been corrected.
